# Neoadjuvant Chemotherapy in Breast Cancer: Review of the Evidence and Conditions That Facilitated Its Use during the Global Pandemic

**DOI:** 10.3390/curroncol28020127

**Published:** 2021-03-24

**Authors:** Tabitha Tse, Sandeep Sehdev, Jean Seely, Denis H. Gravel, Mark Clemons, Erin Cordeiro, Angel Arnaout

**Affiliations:** 1 Department of Surgery, Grand River Hospital, Kitchener Waterloo, ON N2G 1G3, Canada; tabitha.tse@medportal.ca; 2 Division of Medical Oncology, Department of Medicine, University of Ottawa, Ottawa, ON K1H 8L6, Canada; sehdev@mac.com (S.S.); mclemons@toh.ca (M.C.); 3 Division of Breast Imaging, Department of Diagnostic Imaging, University of Ottawa, Ottawa, ON K1H 8L6, Canada; jeseely@toh.ca; 4 Department of Pathology and Laboratory Medicine, University of Ottawa, Ottawa, ON K1H 8L6, Canada; degravel@eorla.ca; 5 Division of General Surgery, Department of Surgery, University of Ottawa, Ottawa, ON K1H 8L6, Canada; ecordero@toh.ca

**Keywords:** breast cancer, neoadjuvant chemotherapy, pandemic, practice change

## Abstract

Practice and behaviour change in healthcare is complex, and requires a set of critical steps that would be needed to implement and sustain the change. Neoadjuvant chemotherapy for breast cancer is traditionally used for locally advanced disease and is primarily advantageous for surgical downstaging purposes. However, it does also offer patients with certain biologic subtypes such as the triple negative or Her2 positive breast cancers the opportunity to improve survival, even in early stage disease. During the height of the pandemic, an opportunity and motivation for the increased use of neoadjuvant therapy in breast cancer was identified. This paper describes the conditions that have supported this practice change at the provider and institutional levels. We also include our own institutional algorithm based on tumor biology and extent of disease that have guided our decisions on breast cancer management during the pandemic. Our processes can be adapted by other institutions and breast oncology practices in accordance with local conditions and resources, during and beyond the pandemic.

## 1. Introduction

Breast cancer is one of the three most common cancers worldwide [1]. The optimal therapy is multimodal and the sequence of therapy takes into account molecular subtype and locoregional tumour burden. As the majority of presentations are of early stage disease, surgical resection is often the primary modality of treatment. However, even with early stage disease, surgery might not be the best initial treatment option for every patient. Neoadjuvant chemotherapy (NAC) was originally introduced to convert patients with inoperable or locally advanced breast cancer into operable disease. Once the benefit of adjuvant chemotherapy in node-positive (and subsequently node-negative) breast cancers was demonstrated, the use of NAC was expanded to include patients who had operable disease [1,2]. Indeed, multiple randomised clinical trials done 30 and 40 years ago confirmed that the survival benefit is equivalent when the same chemotherapy regimen was administered as either neoadjuvant or as adjuvant therapy [3]. The benefits of NAC are well known, and include the ability to downstage the extent of disease in the breast and axilla to increase the rate of breast conservation and avoid complete axillary dissections in patients who have responded well to NAC [4,5,6]. In addition, patients who achieve a pCR can be reassured that they have an excellent distant recurrence-free survival [3,4]. Perhaps most importantly, certain biologic subtypes such as triple negative (TNBC) and Her2 positive (Her2+) patients with residual disease post chemotherapy, who are also the ones at highest risk of recurrence and metastatic disease, will now have the option to receive further effective adjuvant systemic therapies shown to improve disease-free survival [1,7,8,9].

The use of residual disease after NAC to guide further adjuvant treatment represents an important shift in the sequencing of treatment for early-stage TNBC and HER2+ breast cancers. Two landmark clinical trials, namely CREATE-X (UMIN000000843) for residual triple negative [7] and KATHERINE (NCT01772472) for residual Her2+ [8] breast cancers, both demonstrated improved invasive disease-free survival (DFS) with postoperative capecitabine and ado-trastuzumab emtansine (TDM1) respectively. This opportunity for improved invasive DFS, based on residual disease-guided therapy, is lost when patients undergo surgery first. As such, NAC is now considered by the National Comprehensive Cancer Network and St. Gallen International Expert Consensus Panel to be the preferred treatment and the new standard of care for TNBC or Her2+ breast cancer, even for patients who present with stage II and III operable disease [10].

**Figure 1:**
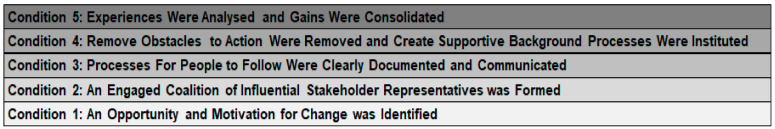
Conditions during the pandemic that facilitated the use of neoadjuvant therapy for breast cancer.

Despite the several advantages of NAC for patients, several studies have shown very low existing rates NAC use in North America, with overall use of 20.2% in the US according to the National Cancer Database [11], and only 5% in a national Canadian study [12]. Interestingly, the recent reports during the COVID-19 pandemic have shown a significant rise in the use of neoadjuvant systemic therapy, both chemotherapy and endocrine therapy, within North America [13,14]. This was and certainly continues to be the case at the Ottawa Hospital Cancer Center, one of the largest cancer centers in the Canada. In a center that treats over 600 invasive breast cancers annually, there was a 200% increase in the use of NAC for TNBC and Her2+ since the first wave of the pandemic, that has continued 7 months later. Such a drastic and rapid change in practice is not common in healthcare. How did this happen? The remainder of the article attempts to understand the conditions that led to the increased utilization of NAC during the pandemic (Figure 1). We also include algorithms based on tumor biology and extent of disease that guided our breast cancer management decisions during the pandemic. Our processes can be adapted by other institutions and breast oncology practices in accordance with local conditions and resources, during and beyond the pandemic.

## 2. Condition #1: An Opportunity and Motivation for Change Was Identified

Following the declaration of the COVID-19 pandemic by the World Health Organization in March 2020, provincial cancer agencies and hospitals rapidly changed the model of cancer care delivery across Canada. The resultant unprecedented pressure on hospital beds and ICU occupancies necessitated rapid re-deployment of staff and capacity towards the management of COVID-19 cases. This led to a deprioritisation of non-emergency clinical services, of which elective cancer surgeries were included [15,16]. For cancer patients, risks related to cancer progression needed to be carefully weighed against the risk of patient and staff exposure to COVID 19, as well as worsened postoperative morbidity in the infected, immunocompromised patient [17]. Elective cancer surgeries in most Canadian hospitals were limited to patients with advanced or rapidly progressing disease, those who would likely suffer a survival disadvantage with surgical delay of >3 months or who were ineligible for systemic therapy [18,19]. These measures were in line with the American College of Surgeons [19], the Society of Surgical Oncology [20] and the European Society of Oncology [21] recommendations of pandemic response to cancer care. In Ontario, the most populated province in Canada, cancer surgeries declined by 30–60% between the months of March to July 2020 compared with the same period in 2019 [18]. Unlike for other kinds of cancers, having neoadjuvant therapy as an option to mitigate the delays in surgery was a major advantage that allowed for treatment initiation during the pandemic, and was welcomed with a sense of relief by most patients and oncologists during an unprecedented time. While there is some evidence of increased risk of chemotherapy toxicity during Covid-19 [21,22], for most patients these risks were considered lower than the perceived increased risks related to surgery in covid positive patients during the height of the pandemic.

## 3. Condition #2: An Engaged Coalition of Influential Stakeholder Representatives Was Formed

Breast cancer treatment is multidisciplinary. The clinical treatment pathways, processes and outcomes for breast cancer patients is a collaborative effort that requires input from diverse specialties. Each specialty acts as change agent to facilitate the entire neoadjuvant process. Clearly, the medical oncologist is a core member of this multidisciplinary team that assesses the patient’s health, goals and clinicopathologic features to determine the appropriateness of NAC systemic therapy in achieving the best oncologic outcomes. Surgeons also play a major role in being gate-keepers for patients undergoing neoadjuvant therapy. They are often the initial specialty to see the patient once diagnosed and a surgeon’s recommendation or preference for care is frequently cited as an important influencer in the decision-making process of a patient [23]. A radiation oncologist should also be consulted upfront in the patient’s course, not only to use the initial presentation determining the extent of postoperative treatment but also to offer salvage radiation if the tumor fails to respond to NAC. Radiologists establish the diagnosis of breast cancer and provide an accurate assessment of the location and extent of the primary breast tumour and determination of the axillary nodal status before and after NAC—critical information for the successful execution of the surgical plan and optimization of the use of subsequent adjuvant radiotherapy. Pathologists can ensure that biomarker results (estrogen and progesterone receptors, and Her2 expression) are available from biopsies at diagnosis. Nurses in the surgical and oncology clinics can help answer any concerns that patients may have regarding the deferral of primary surgical therapy, which for the patients is often the first instinctive method of treatment after diagnosis. At the beginning of the pandemic, Ottawa Hospital Breast Disease Site Group convened influential people from these key stakeholder groups, working at different levels within the organization to form a powerful change coalition that would be working together as a team, requesting their involvement and commitment towards the entire neoadjuvant process. The group met several times to agree on key criteria, indications, and principles of delivery of neoadjuvant therapy during the pandemic. No additional resources or direction was provided by the hospital administration for this. However, providers were highly motivated to figure out solutions to avoid treatment interruption for their breast cancer patients.

## 4. Condition #3. Processes for People to Follow Were Clearly Documented and Communicated

**Figure 2:**
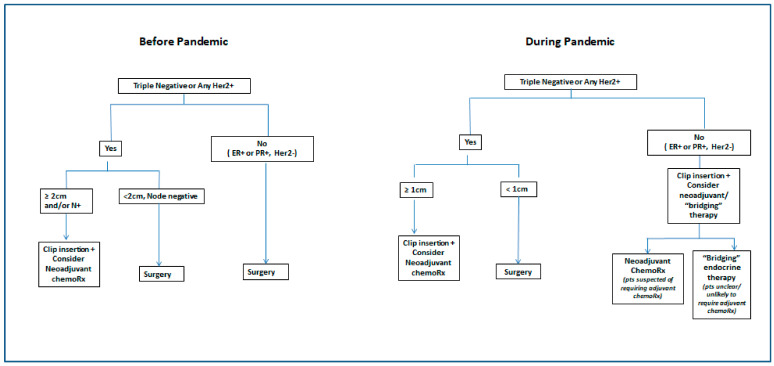
Criteria for neoadjuvant chemotherapy before and during the pandemic at the Ottawa Hospital Cancer Center. “Bridging therapy” refers to endocrine therapy given to temporize surgical treatment. ChemoRX: Chemotherapy; Pts: Patients.

Members of the coalition group created the clear processes and algorithms for people to follow, and communicated it frequently whether in reminder emails, pandemic update emails, and within the multidisciplinary meetings. Figure 2 shows the change in practice at the Ottawa Hospital Cancer Centre. Prior to the pandemic, our institution traditionally limited the use of NAC to patients with locally advanced breast cancer or patients that were TNBC or Her2+ in whom there was an expected significant surgical benefit. This meant that the patient had to have a tumour that was at least 2 cm by palpation or imaging (≥cT2) and/or have 1 or more palpable nodes (N1–3) with a biopsy-proven metastasis. However, if the extent of disease was unclear on imaging, or if there was classical lobular histology, NAC was not recommended in these cases. During the pandemic, however, NAC was initiated in TNBC or Her2+ breast cancers that were 1 cm or larger by clinical exam and/or imaging, whether their axillary nodes were involved or not. It has long been recognized that any systemic therapy which would be offered adjuvantly could reasonably be offered neoadjuvantly, even for smaller tumours, though in light of the benefits outlined above (surgical and prognostic) pre-Covid guidelines strongly advised preoperative systemic therapy mostly for ≥cT2 or N1 cohorts [24]. However, cT1c patients (at least 1 cm) were included in the KATHERINE trial [8] and can be considered for neoadjuvant therapy as per ASCO recommendations [25]. In the Covid-19 era, the additional benefits of avoidance of surgery, decreased impact on staff personal protective equipment (PPE), and on hospital utilization is felt to justify consideration of NAC for smaller tumours, and this approach has been supported by the American College of Surgeons [19] and the Americal Society of Breast Surgeons [22].

In patients who are ER+ and/or PR+, if the decision for benefit of adjuvant and therefore NAC is unclear, the medical oncologist would request Oncotype Dx on the original diagnostic core biopsies [26,27]. Although this is done in our centre during the pandemic, Oncotype Dx is not validated or routinely funded in Ontario for this purpose. However, several studies have shown that NAC may be offered if the Oncotype DX recurrence score is high [24,26], since chemotherapy might be offered postoperatively. The assay is generally feasible if there is at least one core with 2 mm contiguous invasive carcinoma. A high Oncotype DX Recurrence Score is associated with pCR after neoadjuvant chemotherapy based on results of a retrospective analysis of the National Cancer Database of T1-T3 mostly N0 cohort of ER-positive, HER2-negative breast cancer patients [27]. Limited data emerging from a phase II trial also suggest that both pretreatment and post-treatment Recurrence Scores were associated with disease-free survival after neoadjuvant endocrine therapies [26,27]. The Ottawa Hospital and others [28,29] also considered patients with early stage hormone positive disease for bridging endocrine therapy if there was no clear indication forNAC, the disease was not rapidly progressing and surgery was expected to be delayed >8 weeks due to the pandemic (Figure 2).

Final recommendations for surgery depended on the extent of disease at presentation, patient choice, clinical response to NAC, the need for postoperative radiotherapy and genetic testing results, if performed. Surgery after NAC is usually performed 3–6 weeks after the last dose of chemotherapy allow for recovery from toxicity and myelosuppression. Neoadjuvant chemotherapy patients are given highest priority for surgery, with ER- patients more urgent than ER+ or HER2+ patients. Patients with tumours that were borderline unresectable for either breast-conserving therapy or mastectomy were prioritized for surgery ahead of those with a radiographic complete response. Although all guidelines prioritized NAC patients for surgery during the pandemic, they also stipulated that surgery can be safely delayed up to 8 weeks [18,19]. Although some ER+ patients could receive endocrine therapy and HER2+ patients could receive additional antibody therapy to postpone surgery, the safety of this additional delay is unknown. Interestingly, a tool was developed at Massachusetts General Hospital to assist in prioritizing individual breast cancer patients for surgery during the pandemic [30]. It uses a mathematical model that incorporates features of the patient, tumor (imaging, pathologic and genomic features) and delay factors to assign a numerical priority score to each breast cancer patient, with higher scores indicating a higher priority for surgery. The prioritization model obviously has not been validated with long term outcomes but are consistent with published guidelines for prioritization of breast cancer surgeries during the pandemic [31]. Other considerations to determine priority for surgery include overall patient health, COVID-19 risk, and expected hospital resource utilization [32].

Patients receiving tamoxifen who had additional risk factors for perioperative deep vein thrombosis would discontinue it 3 weeks before surgery [33]. If patients were Her2+, trastuzmab was continued up to the day of surgery and be continued shortly thereafter, standard treatment being 18 cycles in 1 year. Although not standard and rarely performed at our hospital prior to the pandemic, consideration for shorter courses of adjuvant trastuzamab treatment can be given to patients at lower risk of relapse and/or higher risk of cardiac toxicity during the pandemic. A recent systematic review and meta-analysis a shorter duration of adjuvant trastuzumab was non-inferior to one year of therapy for DFS in patients with HER2+ disease based on our hazard ratio margin of 1.29, but at the expense of an increase in absolute risk up to 3.9% for 5-year DFS [34]. Thus, potential risks of covid exposure due to repeated hospital visits needs for trastuzamab to be weighed against the higher risk of relapse associated with a shorter duration of treatment [34,35].

## 5. Condition #4: Obstacles to Action Were Removed and Supportive Background Processes Were Instituted

Supportive background organizational processes and structures are necessary to faciliate and align with the strategic goals. At the Ottawa Hospital Cancer Centre, a continuous and iterative process of workflow arrangements in radiology and pathology is necessary to follow the patient management algorithms and enable the seamless management of patients undergoing NAC. In terms of radiology, the increased utilization of neoadjuvant therapy means that radiopaque clips needed to be placed into all invasive cancers (Figure 2). This marker (1) allows the radiologist to have a more accurate identification of the area on post-NAC imaging; (2) acts as the target for localization to guide resection during breast conserving surgery an (3) helps the pathologist to scrutinize that particular area of the resected specimen in search of residual tumor [3,6].

In addition, accommodations were made for the increased use of magnetic resonance imaging (MRI) pre- and post-NAC as it is still the best test to evaluate response to NAC, especially for planned breast conservation post-NAC [3]. In the setting of NAC, a lack of knowledge at presentation of the pathologic axillary nodal status is often of concern [36,37]. Ultrasound-guided core-needle or fine-needle aspiration of a suspicious ipsilateral axillary node with possible clip placement (to facilitate future surgical localization) was performed to document node-positive disease [6].

In terms of pathology, due to the increased utilization of NAC, breast pathologists were expected to examine more post-NAC resection specimens. Careful, systematic evaluation of the post-NAC specimen in the context of clinical and imaging findings is required for accurate diagnosis and evaluation of the response to treatment [38,39], as well as residual-disease guided adjuvant therapy [9]. Correct labelling and indication of the surgical specimen as a post-NAC specimen by the operating room team is crucial. Post-NAC specimens often take longer to evaluate than routine untreated breast cancer specimens, and the extra workload was likely only feasible due to the reduced volume of surgeries that was occurring at the height of the pandemic. Surgeons helped by provision of reports detailing the information about the pretreatment location and size of the tumour for the pathologist. In cases with an excellent response or pCR, a clip marker helps to identify the correct area in the breast for examination, ensures that the appropriate area was excised and can help reduce the number of histologic sections necessary to confidently identifify the site [38,39]. Radiologic, photographic, or pictorial imaging of the sliced specimen are performed to map the tissue sections and reconcile macroscopic and microscopic findings.

Post-NAC changes are complex and several different classification systems for post-NAC specimens are available, each with its own advantages and disadvantages [38,39]. It is essential that pathologists are unified in a common method of histologic assessment of post-NAC specimens. The most commonly cited method of quantification of residual disease is the Residual Cancer Burden (RCB) [40]. It is simple to apply, reproducible, available to anyone for free online (http://www3.mdanderson.org/app/medcalc/index.cfm?pagename=jsconvert3, accessed 5 March 2021) and has validated associations with long-term outcomes (overall survival, event-free survival, and distant relapse-free survival). The residual tumour bed area is initially determined from the macroscopic evaluation, combined with any specimen radiography, and revised after the corresponding tissue sections from that area have been studied under the microscope. The RCB score incorporates gross and microscopic findings in the breast tumour bed and regional lymph nodes. It is calculated from the two-dimensional size of the largest residual tumour bed, the proportion of that residual tumour bed that is invasive cancer, the number of positive lymph nodes, and the largest diameter of the largest nodal metastasis. The formula to calculate RCB combines these variables with adjustment and weighting factors to balance the contributions from different variables and to normalize the distribution of RCB. The continuous RCB score is subdivided in four classes: 0, I, II, and III. An RCB score of 0 corresponds to pCR. The RCB score is prognostic beyond 10 years overall and in phenotypic subgroups [41].

Finally, a discordance in the hormonal receptor and Her2 status can exist between the diagnostic biopsy and post NAC surgical specimens [42]. The clinical utility of reassessing the biomarker status in the surgical specimen may depend on the original biomarker results from the core biopsies taken before NAC. Our pathology department instituted the capacity to perform routine re-testing if the hormone receptors or Her2 status were deemed negative or equivocal on the pretreatment biopsy. This could be done on either the residual primary tumour or residual nodal disease if the latter contained a better representation of residual tumour cells [39,40]. 

## 6. Condition #5: Experiences Were Analysed and Gains Were Consolidated

To keep the momentum and to encourage providers to keep supporting the NAC process, it’s important to not only celebrate short term successes but also to achieve continuous improvement by analysing the success stories and individual experiences at every given opportunity. For many years, the Ottawa Hospital Cancer Centre has used the Communities of Practice (CoP) methodology specifically for this purpose, to not only share collective performance data but also individual insights and experiences. The CoP meetings support knowledge mobilization and collaboration across organizational boundaries, engaging not only various provider specialties but also hospital administrative leaders [43]. During the pandemic, these CoP meetings occurred virtually, and have been well attended. There seemed to be a general need and desire amongst all those who shared the burden of cancer control in the midst of the COVID-19 pandemic, to not only learn together but also from eachother. Our data showed that referrals for newly diagnosed cancers dropped by approximately 40%, likely due to reduced participation in screening mammography programs within the community, As such, the wait times for referral to medical and radiation oncologists for neoadjuvant treatments significantly shortened to within 5 business days. For a 9 month period from March to December 2020, new referrals for NAC increased by 112% compared to the prior year (86 new referrals in 2019 to 96 in 2020), Neoadjuvant endocrine therapy use increased by 365% compared to the prior year (17 new referrals in 2019 to 62 in 2020), as many ER+ patients were placed on “bridging” endocrine therapy during the prolonged wait to surgery.

We and others in oncology specialties have also noted another interesting positive discovery during the pandemic; in that the transition from in-person to virtual multidisciplinary tumor board discussions has improved the ease of attendance and encouraged a greater level of participation at these meetings [44,45]. The virtual format has allowed for greater engagement of breast disease site team members including those at distant sites and from community hospitals. The weekly videoconference format has allowed for screensharing from radiologists and pathologists, and providers have real time access to patient images and records to facilitate real time clinical decision making [41]. In addition, these meetings have allowed for regular updates on the patient management algorithms and new background processes instituted to support them. We feel that having the ability to easily participate in case discussions and having a shared understanding of the indications and benefits of neoadjuvant therapy has also greatly accelerated its uptake and ongoing adaptation during the pandemic.

## 7. Conclusions

Neoadjuvant chemotherapy, traditionally used only for locally advanced breast cancer and primarily advantageous for surgical downstaging purposes, has seen an increased use during the pandemic for earlier-stage disease as well. An opportunity and motivation to facilitate an enhanced neoadjuvant program became apparent at the beginning of the pandemic. The reduced operating room access causing prolonged waits to treatment created a desire for change and engagement by multiple stakeholders. Creating a working group to facilitate change, clearly communicating processes for providers to follow, removing background organizational obstacles and continually consolidating gains are critical steps that would help sustain change. It is important to note that the success of healthcare change requires engagement of all relevant change agent groups, and in this case, the important role that surgeons play in being gate-keepers for patients undergoing neoadjuvant therapy is emphasized. The pandemic can be seen as an opportunity to stimulate a culture of greater acceptance and understanding of the use of neoadjuvant therapy by surgeons, enhancing greater collaboration with radiologists, oncologists, and pathologists. The authors realise the sustainability of a robust neoadjuvant program relies on rapid access to medical oncology and greater resources in pathology (due to the increased workload of reviewing neoadjuvant specimens) and radiology (the increased need for pre- and post-neoadjuvant therapy MRI evaluation). The greater capacity to accommodate the neaodjuvant cases at our site during the pandemic may have been in part, due to the reduction in volumes of breast cancers due to decreased screening and in the reduction of presentation of benign breast disease. It is clear that continued multidisciplinary efforts will be needed to sustain the use of neoadjuvant therapy for early stage breast cancer post pandemic, once screening, volumes of benign breast disease, and operating room access return to normal.
